# Antioxidant Therapies for Ulcerative Dermatitis: A Potential Model for Skin Picking Disorder

**DOI:** 10.1371/journal.pone.0132092

**Published:** 2015-07-13

**Authors:** Nneka M. George, Julia Whitaker, Giovana Vieira, Jerome T. Geronimo, Dwight A. Bellinger, Craig A. Fletcher, Joseph P. Garner

**Affiliations:** 1 Department of Pathology and Laboratory Medicine, Division of Laboratory Animal Medicine, University of North Carolina at Chapel Hill, Chapel Hill, North Carolina, United States of America; 2 Department of Animal Sciences, Purdue University, West Lafayette, Indiana, United States of America; 3 Department of Comparative Medicine, Stanford University, Stanford, California, United States of America; 4 Department of Psychiatry and Behavioral Sciences, Stanford University, Stanford, California, United States of America; Max-Delbrück Center for Molecular Medicine (MDC), GERMANY

## Abstract

Skin Picking Disorder affects 4% of the general population, with serious quality of life impacts, and potentially life threatening complications. Standard psychoactive medications do not help most patients. Similarly, Mouse Ulcerative Dermatitis (skin lesions caused by excessive abnormal grooming behavior) is very common in widely used inbred strains of mice, and represents a serious animal welfare issue and cause of mortality. Treatment options for Ulcerative Dermatitis are largely palliative and ineffective. We have proposed mouse Ulcerative Dermatitis as a model for human Skin Picking Disorder based on similar epidemiology, behavior, and its comorbidity and mechanistic overlap with hair pulling (trichotillomania). We predicted that mouse Ulcerative Dermatitis would be treated by N-Acetylcysteine, as this compound is highly effective in treating both Skin Picking Disorder and Trichotillomania. Furthermore, we hypothesized that N-Acetylcysteine’s mode of action is as a precursor to the production of the endogenous antioxidant glutathione in the brain, and therefore intranasal glutathione would also treat Ulcerative Dermatitis. Accordingly, we show in a heterogenous prospective trial, the significant reduction in Ulcerative Dermatitis lesion severity in mice receiving either N-acetylcysteine (oral administration) or glutathione (intranasal). The majority of mice treated with N-acetylcysteine improved slowly throughout the course of the study. Roughly half of the mice treated with glutathione showed complete resolution of lesion within 2-4 weeks, while the remainder did not respond. These findings are the first to show that the use of N-acetylcysteine and Glutathione can be curative for mouse Ulcerative Dermatitis. These findings lend additional support for mouse Ulcerative Dermatitis as a model of Skin Picking Disorder and also support oxidative stress and glutathione synthesis as the mechanism of action for these compounds. As N-Acetylcysteine is poorly tolerated by many patients, intranasal glutathione warrants further study as potential therapy in Skin Picking, trichotillomania and other body-focused repetitive behavior disorders.

## Introduction

Mice with Ulcerative Dermatitis (UD) can scratch deep skin lesions across large portions of their bodies. UD is a major welfare issue in mice—besides involving painful tissue injury, it is the primary cause of death in aged mice [1], and is a serious confounding experimental factor for the growing number of studies involving aged mice. While UD has been well studied in the veterinary literature for these reasons [2–4], it has recently been proposed as a potential model of Skin Picking Disorder in humans [5]. Skin Picking Disorder is surprisingly common (probably affecting 4% of the general population) [6], but most patients avoid seeking help, often with serious consequences (e.g., 30% of patients had received oral or topical antibiotics, and 5% intravenous antibiotics, to treat secondary infections, prior to seeking psychiatric help)[7]. Despite their enormous impact, psychopharmacological interventions for UD and Skin Picking Disorder have not been established.

Very little is known about Skin Picking in humans that could be used to validate UD unequivocally. We therefore consider UD to be a potential model, based on behavioral and epidemiological similarities. In humans Skin Picking Disorder is highly comorbid with compulsive hair pulling (trichotillomania)[6]. Similarly, mouse strains prone to UD also tend to be prone to ‘barbering’ which has been validated as a model of trichotillomania [8], and the two behaviors share at least some common mechanisms in mice [5]. Epidemiologically, UD is female biased and onsets after sexual maturity. In humans Skin Picking (SP) is also female biased [9], although it tends to onset during puberty. Thus, although UD is not a model for trichotillomania *per se*, trichotillomania (in humans) and barbering (in mice) are useful starting points to approach the problem of Skin Picking Disorder and UD. Although the mode of action is unclear, trichotillomania [10] and Skin Picking Disorder [11] both respond to N-Acetylcysteine (NAC). Ongoing work in our lab suggests a primary role for oxidative stress in barbering and UD [12]. It is unclear whether oxidative stress may be acting peripherally or proprioceptively to drive abnormal grooming behavior (e.g. via itch), or whether oxidative stress is acting centrally in the brain. To address these issues, and to begin to separate these mechanistic possibilities, we treated mice with two leading antioxidant therapies: systemic (oral) NAC, and intranasal glutathione (GSH). We have argued that the efficacy of NAC in treating compulsive hair pulling in humans (trichotillomania), and in mice (barbering), reflects its role as precursor to GSH synthesis in the brain [12]. Therefore, direct intranasal administration of GSH is a further test of this hypothesis.

Originally described in several strains of mice by Stowe in 1971 [4], UD is prevalent in the commonly used C57BL/6 mouse and its sub-strains [1,2,4]. Clinically, mice with UD appear pruritic with regions of alopecia and excoriation, which typically first present in the inter-scapular region. Lesions have a variable progression but often extend to include skin on the face, neck, forelimbs and dorsal-lateral thorax. Those with large granulation tissue beds may heal by scar formation, leading to contracture and impeded mobility [3]. Grossly, lesions range from mild superficial excoriations to deep ulcerations extending through the subcutaneous layers to involve underlying muscle [2]. Histologically, immune complex leukocytoclastic vasculitis has been described [1] along with a mixed inflammatory infiltrate [2]. Chronic wound edges are often hyperplastic and infiltrated secondarily with opportunistic bacteria that result in immune lymphadenopathy and splenomegaly [2]. Unfortunately, these lesions often progress, necessitating humane euthanasia prior to study endpoints [2]. Consequently, not only is UD a welfare issue in mice, but it is a major problem for investigators. UD can result in lost data, scientific aims confounded by the consequences or treatment of UD lesions, and the need to increase animal numbers (and thus cost) in anticipation of attrition of mice to UD, especially in long-term studies using models on C57BL/6 backgrounds [2,4].

Until recently, UD was considered to be an idiopathic dermatitis (i.e. a spontaneously occurring skin lesion, with no known causative agent), and the conspicuous scratching seen in mice with UD was assumed to be secondary to the lesion itself [3]. However we recently reported that mice that develop UD later in life show abnormal grooming and scratching months in advance of the lesion itself [5]. This observation suggests that the ultimate cause of UD is a behaviorally induced mechanical lesion. Indeed, several other authors have made the same point (e.g. [13])–for instance the pathophysiology of UD wounds do not differ from other mechanically induced wounds, and of particular relevance here, oxidative stress pathways in UD lesioned tissue do not differ from those in normal wounds [13]. Indeed, this result suggests that oxidative stress is more likely contributing to the maintenance or triggering of the behavior, rather than a pathological process in the wound itself. Thus, we consider the notoriously multifactorial nature of the disease to reflect the range of factors that can escalate an at-risk animal to self-injurious levels of scratching. At a population level, environmental [2,3], genetic [4], and nutritional [3,5,14] risk factors have all been documented. The profound differences in UD risk between different mouse strains [3], and the strong female bias [2] point to genetic influences. Both UD and barbering are frequently reported unexpected phenotypes in mutant mice [15], many of which involve genes implicated in regulating metabolic rate [e.g., lethal yellow [16], or IRS1 [17]] and oxidative stress [e.g., NF-KappaB [18]]. Furthermore, almost all of the known environmental risk factors for UD can be interpreted in light of their effect on oxidative stress and metabolic rate regulation. For example: seasonal fluctuations in UD [4], high fat diets [14,17], vitamin E in the diet [19], calorie restriction [14], and more complex dietary manipulations [5]. Palliative symptomatic treatments can have some benefit, including ibuprofen [20], and substance P blockade [21–23], but these examples are again consistent with either oxidative stress and/or behavior (via breaking the itch-scratch cycle). However, a consistently effective treatment focused on the underlying mechanism of pathological scratching behavior has not been described.

We have proposed UD as a model of Skin Picking Disorder [5] on the basis of similar epidemiology, behavior, and comorbidity and mechanistic overlap with hair pulling. Similarly other authors have suggested that UD related to excessive or abnormal grooming as an unexpected phenotype in mutant mice may provide useful hints to the genetics of Skin Picking Disorder (the most compelling of which is *SAPAP3*, which is also a known genetic risk factor for Body Focused Repetitive Behaviors including Skin Picking) [6]. Like UD, the study of Skin Picking Disorder is in its early stages. Existing work shows considerable overlap with trichotillomania, both in terms of pathophysiology and treatment response [6]. Only Cognitive Behavioral Therapy shows consistent efficacy in controlled studies of Skin Picking Disorder, while most psychoactive drugs show inconsistent outcomes [6].

NAC, however, was highly efficacious in treating trichotillomania in a randomized double blind placebo controlled trial [10], and open-label case reports suggest the same may be true for Skin Picking Disorder [11]. However, the mechanism of action is unclear: while authors with a psychiatric focus favor subtle interactions with glutamate receptors [10,11], authors with a neurological focus favor oxidative stress as a likely mechanism of NAC efficacy in other disorders [24]. Although these possibilities are not mutually exclusive (not least because GSH influences glutamate transmission via the redox modulatory site on the NMDA receptor), in our opinion the lack of reported glutamatergic side effects in patients treated with NAC argues against a primary role for glutamatergic modulation.

Metabolic oxidative stress is the balance between the production of free radicals as a byproduct of normal glucose metabolism and the ability of cells to scavenge free radicals before they can cause intracellular damage (including lipid peroxidation, interfering with the effective functioning of synapses, damaging DNA and depleting ATP) [25]. The pivotal compound in this balance is Glutathione (GSH). CNS neurons are particularly susceptible to oxidative stress given their high metabolic rate, the low level oxidative neurotoxicity of transmitters such as dopamine, and the inability of many antioxidants to cross the blood brain barrier [25]. Consequently, GSH is the major antioxidant defense against oxidative stress in the brain [25]. GSH synthesis requires cysteine, for which NAC is a major source. Indeed, as NAC is both lipid and water-soluble it can completely compensate for a lack of intracellular cysteine in mice lacking neuronal uptake transporters for cysteine [26].

Accordingly, we have hypothesized that the underlying disease process in barbering (and trichotillomania) may in fact be a failure to buffer oxidative stress during critical periods of brain development, and have shown that standard biomarkers of oxidative stress are elevated 10-fold in barbering mice, and that NAC treatment both cures and prevents barbering in C57Bl/6 mice [12]. Given the overlap between Skin Picking Disorder and trichotillomania in humans, and UD and barbering in mice, we hypothesized that UD might also be a disease of oxidative stress. As a first test of this hypothesis, this study investigates the efficacy of NAC to treat UD in mice. As noted above, if NAC is working by boosting production of GSH in the brain, then intranasal GSH should also be effective in treating UD.

## Materials and Methods

### Ethics Statement

The study protocol was approved by The University of North Carolina at Chapel Hill Institutional Animal Care and Use Committee (IACUC) and all procedures were conducted in accordance with the Guide for Care and Use of Laboratory Animals. Animals requiring humane euthanasia, based on the approved protocol, were euthanized with gaseous carbon dioxide (supplied using a compressed gas cylinder) at the gradual displacement rate of 30% followed by a secondary method of euthanasia.

### Study Design & Enrollment Criteria

The study was performed as a randomized controlled single-blind trial. Mice were recruited from clinical cases detected by caretakers during their daily rounds. The investigator working with each mouse was contacted and given the option of standard veterinary care, or transferring the cage to the study. For social stability, each proband mouse (i.e. the mouse that first presented with UD) was housed with its cagemates throughout the course of the study. Singly housed mice were also accepted. Given the variable nature of the study population, a rich variety of strains (primarily on the C57BL/6 background) were represented from sterile, conventional, breeding and experimental colonies. Using data provided by the original investigators, mice were assigned to one of three treatments at random, while balancing for important confounding variables (sex, strain, age, date of enrollment).

To be enrolled, mice had to receive a diagnosis of UD from the veterinary staff (i.e. areas of dermal ulceration and erythema localized primarily to the face, dorsal neck, and front limbs that cannot be better explained by another diagnosis). Thus animals were excluded if evidence of fighting (lesions noted around the base of tail and perineum) was seen. Similarly if mice had presented (none did) with ectoparasites they would have been excluded. Mice with additional pathology that would not affect UD (alopecia, malocclusions, ocular opacities, etc.) were not excluded. Probands that were enrolled, but that were subsequently removed from the study before at least two measures could be made (either for humane endpoints, or because they were returned to the original investigator), were excluded from statistical analysis (because no assessment of treatment response could be made). Mice with a prior intervention likely to affect UD (e.g., nail trimming) were excluded. Enrolled mice that were currently on treatments for UD were given a 5-day wash out period.

### Housing, Husbandry, and Health Status

Animals were located in 2 buildings distributed among 5 rooms. All animals were housed in polycarbonate individually ventilated cages (Tecniplast, Buguggiate (VA) Italy). All animals received either irradiated corncob ¼ inch bedding (Bed-o-cobs, Anderson, Maumee, Ohio) or cellulose paper bedding (ALPHA-dri, Shephard Specialty Papers, Watertown, TN). Environmental enrichment included at least one or any combination of the following: nestlets (Ancare; Bellmore, NY); mouse house (Tecniplast, VA, Italy); or 3 inch PVC tubing. Cages were changed every other week and spot changed as needed. Mice in one building received reverse osmosis treated water, while mice in the other building received an autoclaved municipal water source. All water bottles were changed weekly. Breeding colony animals were offered *ad libitum* irradiated breeder diet (Harlan Teklad, 2919, Indianapolis, IN) and all others were given a standard irradiated diet ad lib (Harlan Teklad 2920X, Indianapolis, IN). Ventilation rates for cages on the rack were 75 air changes per hour. Mice were maintained on 12:12 light dark cycles, with temperature ranges set between 70F and 74F and humidity between 30% and 70%.

All mice were considered free of the following pathogens by every 4 month bedding sentinel testing: epizootic diarrhea of infant mice (EDIM), Theiler's murine encephalomyelitis virus (TMEV) and GDVII strain of TMEV, Mouse hepatitis virus (MHV), Mycoplasma pulmonis, mouse parvoviruses (MPV and Parvo NS-1), Minute Virus of Mice (MVM), Pneumonia Virus of Mice (PVM) and Sendai virus (serologically), *Radfordia affinis*, *Myobia musculi*, *Myocoptes musculinus*, *Syphacia obvelata*, *Syphacia muris*, *Aspiculuris tetraptera* (by direct observation). Direct sampling for ectoparasites was performed on all enrolled cages of mice (microscopic examination of fur pluck) and found to be negative.

### Humane Endpoints

All enrolled mice were visualized daily during the course of the study for general health and application of study treatments, and dermal changes were documented on all mice (cagemates and proband) every two weeks. Weight and Body Condition Score (BCS)[27] were used in the assessment of health. Mice exhibiting additional clinical signs (including BCS ≤ 2, lethargy, decreased eating or drinking, or indications of systemic illness) were humanely euthanized. Mice showing progression of affected areas exceeding a total surface area of 2cm^2^ on the body and/or neck, 1cm^2^ on the face, or a lesion that extended through all skin layers, were humanely euthanized. If the proband in the cage was humanely euthanized, the cagemates used to maintain social structure were returned to the original investigator and subsequent data was not collected.

### Treatments & Blinding

Faced with a painful and potentially lethal condition, we adopted a ‘current best practice’ design, where all mice with UD (as a proband or having developed UD during the course of the study) received topical palliative treatment, and the treatment groups received either NAC or GSH. All cages had uniform external appearance (water bottles were covered in aluminum foil and no visible treatment information cage side) to keep observers blinded to the treatments.

The ‘None’ control group was treated with a thin film of antibiotic and steroidal ointment (neomycin and polymyxin B sulfates, bacitracin zinc, and hydrocortisone ophthalmic ointment: Akorn Inc., Lake Forrest, IL) applied to all lesions daily for 8 weeks. Based on the extent of the lesion, one to three, 1mm x 15 mm strips of ointment (~ 0.15g maximum) were applied using cotton tipped applicators (9006, Dukal Corporation, Ronkonkoma, NY). Eleven probands were randomized to this control group. Three cagemates in this group developed UD during the course of study. As these mice were treated only with ointment (not experimental antioxidants), they were included in the final analysis as additional ‘None’ controls. In total 11 mice were initially randomized to this group, and further 5 cagemates (3 cagemates of ‘none’ probands, and 2 cagemates of GSH probands) were also added for a total of 16 mice.

The GSH group received 50ul intranasal daily administration of 100mg/ml L-Glutathione reduced (G4251, Sigma Aldrich Corp, Saint Louis, MO) for 8 weeks. Up to 3 attempts were made to place 2 drops on the nostrils of each affected mouse (to reach a target dose of 165 mg/kg). As mice are obligate nose-breathers, this treatment is a simple alternative to aerosol delivery. Dosages of intranasal GSH were based on extrapolated data from human literature [28] and solubility. Bulk GSH was stored, prepared and transported to optimize efficacy (with desiccants, protected from light, and chilled). Thirteen probands were randomized to this treatment group and received treatments daily for the duration of the study. Two untreated cagemates from the GSH group developed UD. As these mice were treated only with ointment they were included in the final analysis as ‘None’ controls.

The NAC group was given N-Acetyl-L-cysteine (A7250, Sigma Aldrich Corp, Saint Louis, MO) in drinking water. Based on a 1-week pilot study (observing weight and level of dehydration) including 9 mice, the highest concentration readily ingested was 2g/250ml. (It is recommended that palatability of NAC be tested before beginning treatment, as there appears to be strain variation in palatability.) Test concentrations were extrapolated from published human data [29]. The average water consumption for C57B6/J mice is 8ml/ 30grams of body weight [30], thus yielding an average daily dosage of 2133mg/kg. Bulk NAC was stored, prepared, transported and maintained cage side to optimize efficacy as practical (with desiccants, protected from light, and chilled). All animals in the cage received treated water. Twelve probands and their cagemates were randomized to this treatment group. No NAC cagemates developed UD during the course of the study.

### UD, Scoring System and Analysis

#### Lesion documentation and verification

Every 2 weeks, a blinded observer removed all mice from the cage individually and examined them thoroughly to document UD lesions. The extent (percentage of body surface area) and severity (depth and/or magnitude) of dermatitis lesions was noted during examination using the topographical ‘mouse maps’ our group has previously reported for scoring barbering [16]. Photographs (dorsal, ventral, lateral and facial views) were taken of all mice at each examination for further reference.

#### Scoring system

Ulcerated lesions were shaded on the map to represent the extent of the lesion and distinct shading colors were chosen to represent lesion severity. A five (5)-point scale, corresponding to 5 distinct shading colors, was used to document the severity of dermatitis lesions (see Table 1). The lesions were categorized with respect to intensity of red color, moisture content of the lesions, predominance of crusts (scabs containing dried serum or blood), severity of skin loss, and health of granulation tissue (connective and vascular tissue found in a healing wound). Crusts ranged from punctate (pinpoint) to predominating in the wound while skin loss ranged from excoriations (superficial scratches) to ulcerations (loss of skin layers of variable depths). Granulation tissue was considered healthy while pink but unhealthy when a deep red color was present. A score of 0 was considered a normal mouse with no obvious lesion or redness noted; 1: was a mild predominantly red lesion, punctuate crust or excoriations were noted but did not predominate; 2: were moderate red lesions with predominant crusts and excoriations, dry healthy granulation tissue and minor partial thickness ulcerations; 3: were marked red moist lesions, no crusts were present, ulceration of skin layers is noted without muscle exposure, unhealthy granulation tissue without active bleeding; 4: was severe, red, and moist with skin abraded to lower levels of the dermis, deep ulcerations that could include muscle exposure and active bleeding was possible. Lesions that were actively bleeding or full thickness requiring humane euthanasia fell into this category.

**Table 1:** Ulcerative Dermatitis Lesion Severity Scale. Lesions were graded for severity on a five point scale as shown, and drawn on a standardized ‘mouse map’. The UD score for each mouse was calculated as the area of the lesion weighted by the severity.

0	A normal mouse with no obvious lesion or redness noted.
1	Mild. Predominantly red lesion, punctuate crust or excoriations were noted but covered less than 50% of the lesion.
2	Moderate. Red lesions with crusts and excoriations covering at least 50% of the lesion, dry healthy granulation tissue and/or minor partial thickness ulcerations.
3	Marked. Red moist lesions, no crusts were present; ulceration of skin layers is noted without muscle exposure, and/or unhealthy granulation tissue without active bleeding.
4	Severe. Red and moist lesions with skin abraded to lower levels of the dermis, deep ulcerations that could include muscle exposure and active bleeding was possible.

The resulting maps were processed in Fiji, an image-processing program [31]. The total lesion area (as a percentage of the total body area), scaled by the severity of the lesion, was calculated, following the methods previously described for barbering [16].

### Data Processing and Statistical Analysis

Mice were coded as probands (the individual which first caused the cage to be entered into the study) or cagemates, and by the experimental drug administered (GSH, NAC or None). All mice given the designation of ‘None’ received standard topical treatment but no experimental drug. The cagemates that developed UD during the course of the study were assigned as described above. To test for potential differences in UD lesion score at baseline, we performed a one-way ANOVA using a GLM on the log-transformed UD score, selecting the week 0 data for probands only.

To test the effect of treatment on UD score, we performed a REML mixed model repeated measures analysis, including all the animals that showed UD lesions during the course of the study. Subject was nested within Drug as a random effect, and the fixed effects of drug, day of observation, and their interaction modeled. A key advantage of this method is that it is extremely robust to missing and unbalanced data (e.g., caused by animals being removed from study prematurely). Furthermore, by including Subject, all the known and unknown confounding features of that animal (e.g., sex, age, *etc*.) are controlled for by the analysis. The Drug*Day interaction specifically tests whether the rate of improvements (or worsening) differs between treatment. As UD Lesion Score was log-transformed, rate of change can be expressed in units of fold-change (or percentage change) per unit time. *Post hoc* tests were performed as Bonferroni-corrected planned contrasts.

Initial inspection of the data suggested that GSH *versus* NAC treated animals might be responding differently, whereby most NAC treated animals responded, albeit slowly; but GSH treated animals either responded rapidly, or not at all. Furthermore, within GSH treated animals, the non-responders tended to have more severe lesions at the start of the study. To test this impression we adopted two approaches. First, we performed a logistic regression predicting survival, controlled for gender, age, and testing drug, initial UD lesion score and their interaction. As almost all animals in the ‘None’ treatment did not recover, including these animals lead to quasi-complete separation of the model and an unstable REML solution. Therefore this analysis was ultimately conducted on only the GSH and NAC treated animals. *Post hoc* tests were performed as planned contrasts. Second, we performed a parametric survivorship analysis, right-censored for both the end of the study, and early removal from the study, using the same model. While the resulting analysis was highly significant the REML estimates showed quasi-complete separation of the model, and so could not yield useful estimates of survival and time-to-cure. Therefore, we performed a simpler analysis, focusing only on the individuals who were cured (i.e. had lesion scores of zero by the end of the study), and used a GLM to test whether the time to cure differed between GSH and NAC treatments. All tests were performed in JMP Pro 11 for Windows, with additional *post hoc* tests performed using identical models in SAS 9.4 for Windows for convenience as required.

## Results

### Baseline UD lesion scores

Probands did not differ in baseline UD score (F_2,33_ = 0.0215; P = 0.9788) (Figure A in S1 Dataset). However in the full analysis, where the ‘None’ group contained 5 cagemates that were initially asymptomatic, these individuals caused the ‘None’ group to have a lower mean UD lesion score at Week 0, though this artifact is eliminated by Week 2.

### Mean rate of change in UD lesion score

**Fig 1:**
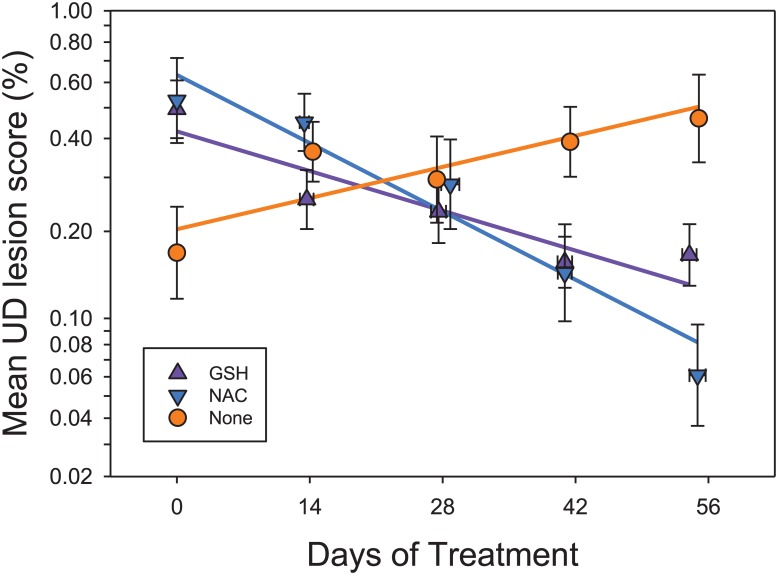
Mean UD lesion score as a function of time. Probands did not differ in mean score at week 0. However, cagemates of probands who later developed UD are included, reducing the mean score for ‘None’ animals at week 0. Data are shown as the arithmetic mean +/- SE of the lesion scores corrected for subject, averaged at each week (horizontal error bars show the SE of the day of observation). Post-hoc tests of the slopes are shown in Fig 2.

**Fig 2:**
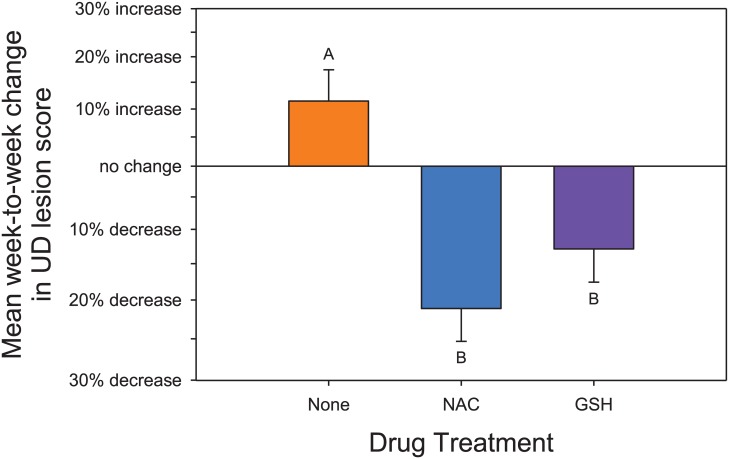
Weekly change in % of lesion score. The data in Fig 1 is shown as the mean rate of change for each treatment. Data are figured as the % change per week in Lesion score. Means with the same subscript do not differ significantly. Both NAC and GSH treated animals showed a significant decrease in lesion scores over time.

Change in UD lesion score differed significantly by time (Drug*Day interaction: F_2,112.7_ = 11.17; P < .0001) (Fig 1, Figure B in S1 Dataset). *Post hoc* tests revealed that the mean rate of change in UD lesion score differed between NAC and None, GSH and None, but not GSH and NAC; furthermore, both NAC and GSH showed significant decreases over time, while None animals showed a non-significant increase (Fig 2).

### Survivorship and time to cure

**Fig 3:**
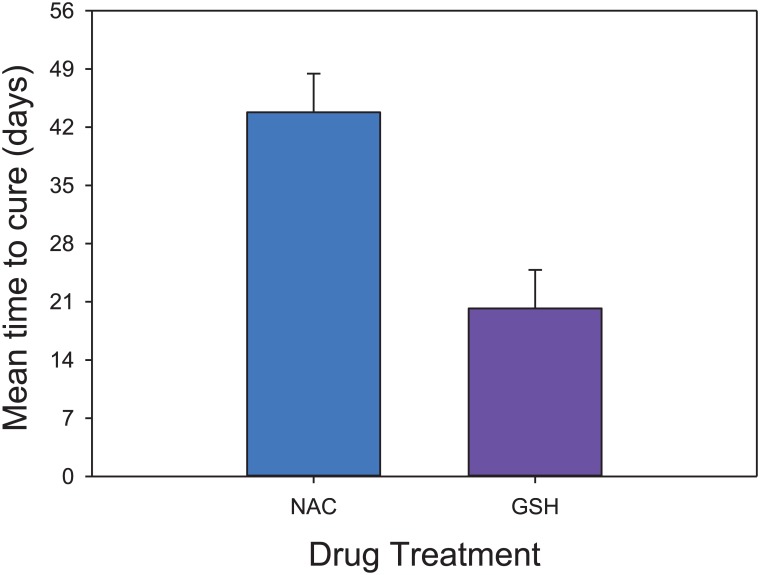
Mean time to Cure. For animals that were completely cured (i.e. had final UD lesions scores of zero), GSH achieved this outcome significantly faster than NAC.

**Fig 4:**
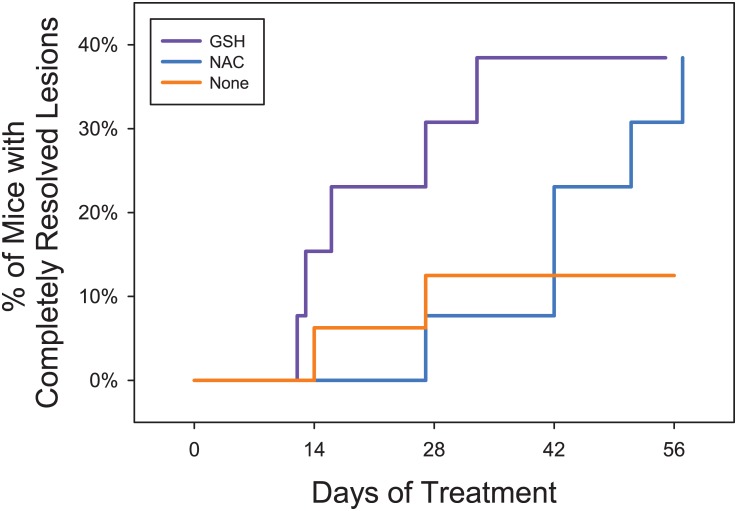
Percentage of Mice Cured Over Time. The raw event data behind Fig 3 are shown. Euthanasia and cure events cannot be show on the same graph, however the final percentage of animals cured also reflects the cumulative proportion of animals euthanized by the end of the study (i.e. every non-cured animal was euthanized at some point during the study according to our human endpoints).

These mean changes mask an important feature of the data—almost all NAC animals improved (albeit slowly and with roughly 40% being fully cured); whereas the GSH treated animals showed an all-or-nothing response, where roughly 40% of the GSH treated animals resolved within 2–4 weeks, while the remainder did not improve. The GLIM logistic regression confirmed this observation (Figure C in S1 Dataset). Thus, the probability of being cured was strongly predicted by the Drug*Baseline UD interaction (LR ChiSq = 7.615; P = 0.0058). The chance of being cured was unaffected by baseline UD for NAC treated animals (P = 0.395), but increased as a progressive function of lower baseline UD scores in GSH treated animals (P = 0.0028). Furthermore, focusing just on the animals which were cured, the time to cure was significant shorter in GSH treated animals than NAC treated animals (F_1,8_ = 12.93; P = 0.0070) (Fig 3). A time to effect curve, including the control animals in the comparison, shows the percentage of mice cured over time and highlights the fact that animals in the “None” treated group rarely recovered (Fig 4, Figure D in S1 Dataset).

## Discussion

These data show that both oral N-Acetylcysteine and intranasal glutathione can treat Ulcerative Dermatitis, a potential model of Skin Picking Disorder. This finding has several important implications. First, this result provides further validation for UD as a model of Skin Picking Disorder. Second, the effectiveness of intranasal GSH sheds further light on the potential mechanism of action of both compounds. And, third, these results have important implications for treatment of both UD and Skin Picking Disorder.

### Implications for the model

Although NAC has been reported to prevent UD in mice being treated for barbering, and has been reported to treat barbering in mice [12], these data are the first to show that NAC or GSH can cure UD lesions in mice. NAC reduced symptoms significantly in 56% of trichotillomania patients in a randomized double blind placebo controlled trial [10]; completely resolved Skin Picking Disorder in a case report [11]; and significantly reduced lesions in 100%, and completely resolved Skin Picking symptoms in 71%, of adults and children with Prader-Willi syndrome in an open-label trial [32]. We are unaware of any human trials of GSH in trichotillomania or Skin Picking Disorder. Nevertheless, the similar treatment response to NAC is one additional piece of evidence validating UD as an animal model of Skin Picking Disorder.

Similar to human trials, most proband mice receiving treatment with NAC showed some reduction in the UD lesion score during the 8-week study regardless of initial lesion severity, with roughly 40% resolving completely (compared with 71% in humans) [32]. This lower cure rate might reflect the fixed dosage that the mice received throughout the experimental period and the shorter duration than the 12 weeks described in human investigations. Studies testing treatment effect of NAC on trichotillomania and Skin Picking Disorder use gradually increasing dosages from 450mg/day [32] to 1200mg twice daily, and this upward dosage adjustment (over a 4 to 12 week period) is often necessary to achieve full resolution in humans [10,11]. The use of humane endpoints could also lead to an underestimate of potential cure rates. Lesion severity dictated euthanasia in many of our non-responders, and these mice may have responded given more time or a higher dose (though our pilot work suggests that, in practice, tested strains of mice did not find higher doses palatable and strain variation in palatability may be noted at the dosage used in the study).

### Implications for mode of action

Previous work in trichotillomania and Skin Picking Disorder in both humans and animals has not expressly tested whether NAC’s effects are due to the compound directly, or as a precursor to GSH; nor whether NAC’s effects are due to action at the periphery, or in the brain. The efficacy of GSH speaks to the first point. GSH is the primary antioxidant used in the brain [25], and intranasal GSH has proven efficacious in several diseases involving oxidative damage (such as Parkinson’s Disease) [33]. Combined with the evidence for elevated oxidative stress seen in barbering mice [12], the most parsimonious explanation is that at least some of NAC’s efficacy is due to GSH synthesis. This in turn argues against a general glutamatergic pathogenesis, and instead for a general role of metabolically induced oxidative stress. The prophylactic effect of NAC in both barbering and UD further supports this notion [12].

The differing pattern of response with GSH *versus* NAC treatment raises a number of possibilities. Oral administration is complicated by the ease of absorbance (or transport) from the gut, degradation by hepatic first pass metabolism, scavenging and metabolism by other organs (which is a particular issue for antioxidants like NAC and GSH), potential side effects in other organ systems, excretion, and uptake across the blood-brain barrier [34]. Although intranasal administration does not exclusively deliver a compound to the brain (e.g., due to ingestion via post-nasal drip), it does eliminate or mitigate many of these problems [34]. Accordingly, in the current experiment, GSH treated mice that showed complete resolution did so within 2–4 weeks of treatment, while NAC treated mice took roughly twice as long. This observation suggests that the primary site of therapeutic action is in the brain. This rapid response has not been described in human treatment of trichotillomania or Skin Picking Disorder with NAC.

At the same time, in the current experiment, GSH treated mice that did not respond were more severe at baseline and showed no improvement, while most NAC treated mice showed at least some improvement regardless of initial severity. One possible interpretation of this pattern of results is that both CNS and somatic metabolism are contributing to oxidative stress in these animals. In which case orally administered NAC is mitigating both sources of redox imbalance, while intranasal GSH is primarily alleviating CNS oxidative stress, and may not (at the doses given) be able to compensate for a redox burden with strong somatic contributions. Consistent with this interpretation nesting material, which reduces metabolic rate in mice [35], also prevents barbering [36].

Prolonged oxidative stress could force affected neurons into apoptosis, or quiescence. For NAC and GSH to be able to resolve UD, the affected neurons are more likely to be quiescent, and thus able to resume normal function with the resolution of oxidative stress. RNA-seq data from barbering mice shows evidence of quiescence, not apoptosis [12]. Nevertheless, the lack of response to GSH in particularly severe UD animals could reflect apoptotic damage. Alternatively, the dose of GSH may simply have been insufficient. A dose-response study would help tease apart these possibilities.

### Implications for treatment

A variety of anecdotal treatments (the majority being topically applied) have been used to mice with UD [2] in an attempt to resolve this condition and address the welfare concern it creates. The use of topical antibiotic and steroid preparations is based on the fact that mixed inflammatory infiltrates and secondary bacterial infections are common histological findings in UD lesions [2]. These palliative treatments are usually ineffective, and accordingly in the current experiment, mice in the ‘none’ group showed a non-significant rise in UD lesion scores. Furthermore, topical treatments can confound results in various research fields. For instance, the use of long-term steroids (to mitigate pain and inflammation) can delay wound healing. Similarly, in Skin Picking Disorder, picking behavior can cause skin infections, septicemia, cellulitis, and scarring that necessitates palliative treatments such as antibiotics, corrective surgery or skin grafts, but effective treatment requires behavioral or psychopharmacological intervention [37][6].

Formal studies of NAC efficacy in Skin Picking Disorder are currently limited to open-label case reports [11]. Thus, the current data provide support for the current use of NAC to treat Skin Picking Disorder. We have previously reported that NAC can prevent UD in cagemates of mice being treated for barbering [12], and that pattern was also seen in the current study, although the numbers of non-treated animals that developed UD during the current study was too small for analysis. Nevertheless, given previous findings, NAC might be a useful prophylactic in at-risk individuals (e.g., siblings or children of existing patients). Furthermore, these data suggest that intranasal GSH might also help Skin Picking Disorder. Although not well documented in the literature, NAC is often poorly tolerated by trichotillomania and Skin Picking Disorder patients. NAC is a mucolytic, and so can induce gastrointestinal side effects; and, anecdotally, patients often complain of an intolerable “rotten egg” smell or taste. NAC’s mucolytic action can also lead to contraindication in some patients. Intranasal GSH does not suffer these drawbacks. A survey of intranasal GSH use in humans noted that the 86% found the treatment comfortable, although 12% reported experiencing adverse effect that generally related to headaches and nasal irritation [38]. Thus, intranasal GSH may be particularly useful as an alternative treatment, particularly in patients that do not respond to NAC, or who cannot tolerate this treatment. As a result, treatment trials of intranasal GSH in trichotillomania and Skin Picking Disorder are a high priority for future work.

### Limitations

The main limitations of this study stems from its prospective nature. The mice were heterogeneous with respect to sex, age, and genetics. While this is often viewed as a confound in animal studies, heterogeneous populations actually improve translational validity given suitable statistical analyses (as employed here)[39]. Again, given the fact that this was the first study of its kind, we chose doses given existing human and animal literature. As the data show, this was sufficient to show an effect of these compounds, but these results also underline the need for future dose-response studies, particularly for GSH. While intranasal GSH administration points towards a central role of oxidative stress, this remains a hypothesis to be tested (for instance, by examining biomarkers of oxidative stress, and by using labeled GSH to confirm its site of action in the brain). Finally, in future studies, the use of whole body PCR swab analysis for dermal parasites would be a nice supplemental feature to the microscopic examination of fur pluck we used.

### Conclusion

The positive response of mice UD lesions to both NAC and GSH provides evidence of predictive validity of UD as a potential model of Skin Picking Disorder, and provides treatment options for mice suffering from this severe health condition. The use of topical antimicrobial and steroid compounds is not supported as a sole therapy for mouse UD. These data lend support to the role of oxidative stress in both Skin Picking Disorder and UD, and encourage investigation of intranasal GSH as a novel therapy for human Skin Picking Disorder.

## Supporting Information

S1 Dataset SAS scripts for supplementary data. The raw data for each of the analyses are presented. Baseline severity difference (probands only) (**Figure A in S1 Dataset**), Repeated measures analysis of change in lesion severity (**Figure B in S1 Dataset**). Logistic regression of survivorship (**Figure C in S1 Dataset**). Time to cure (**Figure D in S1 Dataset**). Each data set is given as a SAS code for the data itself, and the equivalent analysis to that performed in JMP (and reported in the text). Data are presented in SAS format as this is a simple text format. The data and code were generated as direct exports from JMP, and additional SAS code added as needed (for instance, JMP does not export code for post-hoc tests). Note, however, that SAS rounds to less precision than JMP, and can give slightly different results, especially for REML methods.
(DOCX)
