# Supplementary material for: Antioxidant Therapies for Ulcerative Dermatitis: A Potential Model for Skin Picking Disorder
Source: PLoS One. 2015 Jul 13;10(7):e0132092. doi: 10.1371/journal.pone.0132092 (PMC4500395; doi:10.1371/journal.pone.0132092)
Supplement: S1 Dataset — The raw data for each of the analyses are presented. Baseline severity difference (probands only) (Figure A in S1 Dataset), Repeated measures analysis of change in lesion severity (Figure B in S1 Dataset). Logistic regression of survivorship (Figure C in S1 Dataset). Time to cure (Figure D in S1 Dataset). Each data set is given as a SAS code for the data itself, and the equivalent analysis to that performed in JMP (and reported in the text). Data are presented in SAS format as this is a simple text format. The data and code were generated as direct exports from JMP, and additional SAS code added as needed (for instance, JMP does not export code for post-hoc tests). Note, however, that SAS rounds to less precision than JMP, and can give slightly different results, especially for REML methods. (DOCX) [file pone.0132092.s001.docx]

# Supplementary data

The raw data for each of the analyses are presented below. Each data set is given as a SAS code for the data itself, and the equivalent analysis to that performed in JMP (and reported in the text). Data are presented in SAS format as this is a simple text format. The data and code were generated as direct exports from JMP, and additional SAS code added as needed (for instance, JMP does not export code for post-hoc tests). Note, however, that SAS rounds to less precision than JMP, and can give slightly different results, especially for REML methods.

# Figure A - Do mice differ in baseline severity (probands only)?

**DATA** Proband_Baseline; INPUT Drug &$16. log10_UD; Lines;

NAC **0.138870043723654**

NAC -**0.440622789459979**

None -**0.549818755236789**

GSH -**0.969324815747695**

GSH -**0.997394132223973**

None -**0.775285653739941**

GSH **0.136746117447644**

NAC -**0.0830103469135702**

None **0.325443309405612**

NAC -**0.0691092458944324**

NAC **0.329560166971662**

GSH -**0.482838860107672**

None **0.247082397451844**

NAC -**0.18170956308693**

GSH -**0.500945534428269**

NAC -**0.560289841325451**

GSH **0.569871179150474**

NAC **0.398846389947336**

GSH -**0.204458028430609**

None **0.935721087067092**

GSH **0.205520835167247**

None -**0.997394132223973**

None -**0.409576126480923**

NAC -**0.741833049075868**

GSH -**0.758237334821539**

NAC -**0.942960062932372**

None **0.216290015502478**

GSH **0.205520835167247**

GSH -**0.775285653739941**

None -**1.69897000433602**

NAC -**0.616804171138775**

None **0.1281449918352**

GSH **0.103573969061506**

NAC -**0.380601605369128**

None -**0.851080560077292**

GSH -**0.286471993543469**

;

**RUN**;

**PROC** **GLM** DATA=Proband_Baseline ALPHA=**0.05**;

CLASS Drug;

MODEL log10_UD = Drug;

**RUN**;

# Figure B - Repeated measures analysis of change in lesion severity

**DATA** Mean_rate_of_change; INPUT UniqueMouseID &$16. Drug &$16. log10_UD Day; Lines;

**03**-M2 NAC **0.138870043723654** **0**

**03**-M2 NAC -**0.297904825390049** **16**

**03**-M2 NAC -**0.549818755236789** **33**

**03**-M2 NAC -**1.69897000433602** **42**

**03**-M2 NAC -**1.69897000433602** **56**

**04**-M2 NAC -**0.440622789459979** **0**

**04**-M2 NAC -**0.654617314171501** **16**

**04**-M2 NAC -**0.894595071560488** **33**

**04**-M2 NAC -**1.69897000433602** **42**

**04**-M2 NAC -**1.69897000433602** **57**

**05**-M3 None -**1.69897000433602** **0**

**05**-M3 None -**0.997394132223973** **16**

**05**-M4 None -**0.549818755236789** **0**

**05**-M4 None -**0.139502689870799** **16**

**06**-M1 GSH -**0.969324815747695** **0**

**06**-M1 GSH -**1.69897000433602** **16**

**06**-M1 GSH -**1.69897000433602** **42**

**06**-M2 None -**1.69897000433602** **0**

**06**-M2 None -**0.297904825390049** **16**

**06**-M2 None **0.00607615724974619** **42**

**08**-M1 GSH -**0.997394132223973** **0**

**08**-M1 GSH -**1.69897000433602** **33**

**08**-M1 GSH -**1.69897000433602** **42**

**08**-M1 GSH -**1.69897000433602** **57**

**09**-M1 None -**0.775285653739941** **0**

**09**-M1 None -**0.539594200615932** **43**

**09**-M1 None -**0.604897639902608** **56**

**09**-M2 None -**1.69897000433602** **0**

**09**-M2 None -**0.366808415814235** **43**

**09**-M2 None -**0.366808415814235** **56**

**10**-M2 GSH **0.136746117447644** **0**

**10**-M2 GSH **0.157531951985503** **19**

**11**-M4 NAC -**0.0830103469135702** **0**

**11**-M4 NAC -**0.34690677866014** **16**

**11**-M4 NAC -**0.519840082312665** **33**

**11**-M4 NAC -**0.894595071560488** **42**

**11**-M4 NAC -**1.69897000433602** **57**

**12**-M3 None **0.325443309405612** **0**

**12**-M3 None **0.110414197935439** **14**

**15**-M1 NAC -**0.0691092458944324** **0**

**15**-M1 NAC **0.252008702801491** **13**

**16**-M1 NAC **0.329560166971662** **0**

**16**-M1 NAC **0.1281449918352** **13**

**16**-M1 NAC -**1.69897000433602** **27**

**17**-M2 GSH -**0.482838860107672** **0**

**17**-M2 GSH -**0.00855014093240761** **13**

**18**-M1 None **0.247082397451844** **0**

**18**-M1 None **0.117148360187577** **13**

**18**-M1 None -**0.474060963309925** **27**

**18**-M1 None -**0.218701955208002** **41**

**22**-M3 NAC -**0.18170956308693** **0**

**22**-M3 NAC -**0.424822085662098** **13**

**22**-M3 NAC -**0.500945534428269** **27**

**22**-M3 NAC -**0.500945534428269** **41**

**23**-M1 GSH -**0.500945534428269** **0**

**23**-M1 GSH -**1.69897000433602** **13**

**23**-M1 GSH -**1.69897000433602** **27**

**23**-M1 GSH -**1.69897000433602** **41**

**23**-M1 GSH -**1.69897000433602** **54**

**23**-M2 None -**1.69897000433602** **0**

**23**-M2 None -**1.69897000433602** **13**

**23**-M2 None -**0.851080560077292** **27**

**23**-M2 None -**0.741833049075868** **41**

**23**-M2 None -**0.43265058295478** **54**

**25**-M2 NAC -**0.560289841325451** **0**

**25**-M2 NAC **0.201870936705314** **13**

**25**-M2 NAC **0.103573969061506** **28**

**25**-M2 NAC **0.0895615668108325** **42**

**25**-M2 NAC -**0.116016852227225** **54**

**27**-M3 GSH **0.569871179150474** **0**

**27**-M3 GSH **0.572220934350779** **13**

**27**-M3 GSH **0.543982690613419** **27**

**27**-M3 GSH **0.497388705709504** **42**

**27**-M3 GSH **0.64072968472234** **55**

**28**-M3 NAC **0.398846389947336** **0**

**28**-M3 NAC **0.130311295613681** **13**

**28**-M3 NAC **0.0339290881252805** **28**

**20**-M1 GSH -**0.204458028430609** **0**

**20**-M1 GSH -**0.151739720426263** **10**

**20**-M1 GSH **0.00894322181364505** **25**

**20**-M1 GSH -**0.00558520862396215** **36**

**20**-M1 GSH -**0.560289841325451** **51**

**24**-M1 None **0.935721087067092** **0**

**24**-M1 None **0.984613638163069** **16**

**24**-M1 None **1.02222642813533** **27**

**29**-M2 GSH **0.205520835167247** **0**

**29**-M2 GSH **0.205520835167247** **16**

**29**-M2 GSH -**0.0330277736201323** **28**

**30**-M2 None -**1.32913029079336** **0**

**30**-M2 None -**1.13232895814037** **14**

**30**-M2 None -**1.69897000433602** **27**

**30**-M2 None -**1.69897000433602** **40**

**30**-M2 None -**1.69897000433602** **55**

**30**-M3 None -**0.997394132223973** **0**

**30**-M3 None -**1.69897000433602** **14**

**30**-M3 None -**1.69897000433602** **27**

**30**-M3 None -**1.69897000433602** **40**

**30**-M3 None -**1.69897000433602** **55**

**31**-M1 None -**0.409576126480923** **0**

**31**-M1 None -**0.62904635322233** **16**

**31**-M1 None -**0.560289841325451** **28**

**31**-M1 None -**0.195215168975737** **41**

**31**-M1 None **0.119370074742266** **55**

**32**-M1 NAC -**0.741833049075868** **0**

**32**-M1 NAC -**1.69897000433602** **11**

**32**-M1 NAC -**1.09446655468838** **25**

**32**-M1 NAC -**1.09446655468838** **36**

**32**-M1 NAC -**1.69897000433602** **51**

**33**-M1 GSH -**0.758237334821539** **0**

**33**-M1 GSH -**1.32913029079336** **15**

**33**-M1 GSH -**1.69897000433602** **27**

**33**-M1 GSH -**1.69897000433602** **40**

**33**-M1 GSH -**1.69897000433602** **55**

**34**-M1 NAC -**0.942960062932372** **0**

**34**-M1 NAC -**0.654617314171501** **13**

**34**-M1 NAC -**0.321715049872737** **26**

**34**-M1 NAC -**1.02740375210612** **40**

**34**-M1 NAC -**1.21967610876333** **56**

**35**-M1 None **0.216290015502478** **0**

**35**-M1 None **0.631287800050248** **13**

**36**-M1 GSH **0.205520835167247** **0**

**36**-M1 GSH **0.121580481523594** **12**

**36**-M1 GSH **0.59430287624554** **25**

**37**-M1 GSH -**0.775285653739941** **0**

**37**-M1 GSH -**0.604897639902608** **11**

**37**-M1 GSH -**0.582021265691168** **27**

**37**-M1 GSH -**0.582021265691168** **41**

**37**-M1 GSH -**0.360072693834054** **52**

**38**-M1 None -**1.69897000433602** **0**

**38**-M1 None **0.447276365216301** **12**

**38**-M1 None **0.538107832008018** **28**

**39**-M1 NAC -**0.616804171138775** **0**

**39**-M1 NAC -**0.280866495810837** **12**

**39**-M1 NAC **0.0366185996728637** **28**

**39**-M1 NAC -**0.160093594197843** **42**

**39**-M1 NAC -**0.309646783416573** **53**

**40**-M2 None **0.1281449918352** **0**

**40**-M2 None -**0.0298915525328853** **54**

**42**-M1 GSH **0.103573969061506** **0**

**42**-M1 GSH **0.190733504323134** **14**

**42**-M1 GSH -**0.143543509678765** **29**

**42**-M1 GSH -**0.448744080196334** **43**

**42**-M1 GSH -**0.243531898860804** **54**

**43**-M1 NAC -**0.380601605369128** **0**

**43**-M1 NAC -**0.292150789293305** **12**

**44**-M1 None -**0.851080560077292** **0**

**44**-M1 None -**1.02740375210612** **13**

**44**-M1 None -**1.17381027094998** **28**

**44**-M1 None -**1.17381027094998** **42**

**45**-M1 GSH -**0.286471993543469** **0**

**45**-M1 GSH -**1.69897000433602** **12**

;

**RUN**;

**PROC** **MIXED** ASYCOV NOBOUND DATA=Mean_rate_of_change ALPHA=**0.05**;

CLASS UniqueMouseID Drug;

MODEL log10_UD = Drug Day Drug*Day/ SOLUTION DDFM=KENWARDROGER;

RANDOM UniqueMouseID(Drug ) / SOLUTION ;

* Estimates provide the rates of change, SE, and tests in Figure 3;

ESTIMATE 'GSH rate of change' day **1** Drug*day **1** **0** **0**;

ESTIMATE 'NAC rate of change' day **1** Drug*day **0** **1** **0**;

ESTIMATE 'NONE rate of change' day **1** Drug*day **0** **0** **1**;

* Contrasts test differences between each rate of change ;

CONTRAST 'GSH vs NAC rate of change' drug*day **1** -**1** **0**;

CONTRAST 'GSH vs NONE rate of change' drug*day **1** **0** -**1**;

CONTRAST 'NAC vs NONE rate of change' drug*day **0** **1** -**1**;

**RUN**;

# Figure C - Logistic regression of Survivorship

**DATA** UD_survival_LogReg; INPUT Gender &$16. Drug &$16. AgeAtDay0 Log10_Week0_UD SurvivalEvent &$16.; Lines;

M NAC **303** **0.132515009323913** Cured

F NAC **346** -**0.465266184024774** Cured

M GSH **953** -**1.05889300781587** Cured

F GSH **347** -**1.09365511407509** Cured

M GSH **313** **0.130359697297778** CensoredSick

F NAC **326** -**0.0936551140750858** Cured

M NAC **485** -**0.0794146749604755** CensoredSick

M NAC **464** **0.32547419366689** Cured

M GSH **472** -**0.510078528441137** CensoredSick

M NAC **655** -**0.195112754833863** CensoredSick

F GSH **632** -**0.529383683636523** Cured

F NAC **104** -**0.5930527635059** CensoredSick

M GSH **659** **0.567526329371533** CensoredSick

F NAC **492** **0.395365363944284** CensoredSick

F GSH **214** -**0.218593850683386** CensoredSick

F GSH **539** **0.200075642847396** CensoredSick

M NAC **226** -**0.792625118411105** Cured

F GSH **92** -**0.811108524105118** Cured

F NAC **420** -**1.02670832444447** CensoredSick

M GSH **407** **0.200075642847396** CensoredSick

M GSH **345** -**0.830413679300504** CensoredSick

M NAC **829** -**0.654322420244823** CensoredSick

F GSH **567** **0.0966765840952058** CensoredSick

M NAC **1120** -**0.401984348480566** CensoredSick

;

**RUN**;

**PROC** **GENMOD** DATA=UD_survival_LogReg desc;

CLASS Gender Drug;

MODEL SurvivalEvent = AgeAtDay0 Log10_Week0_UD Gender Drug Log10_Week0_UD*Drug / DIST=Binomial LINK=Logit type3;

CONTRAST 'GSH baseline effect test' Log10_Week0_UD **1** Log10_Week0_UD*Drug **1** **0**;

CONTRAST 'NAC baseline effect test' Log10_Week0_UD **1** Log10_Week0_UD*Drug **0** **1**;

**run**;

# Figure D - Time to cure

**DATA** UD_time_to_cure; INPUT Drug &$16. DaysToEvent; Lines;

NAC **42**

NAC **42**

GSH **16**

GSH **33**

NAC **57**

NAC **27**

GSH **13**

NAC **51**

GSH **27**

GSH **12**

;

**RUN**;

**PROC** **GLM** DATA=UD_time_to_cure ALPHA=**0.05**;

CLASS Drug;

MODEL DaysToEvent = Drug;

LSMEANS drug/ STDERR;

**RUN**;
